# Role of Linkers between Zinc Fingers in Spacing Recognition by Plant TFIIIA-Type Zinc-Finger Proteins

**DOI:** 10.1155/2012/848037

**Published:** 2011-11-03

**Authors:** Setsuko Fukushima, Michiteru Yoshida, Hiroshi Takatsuji

**Affiliations:** ^1^Disease Resistant Crops Research Unit, Division of Plant Sciences, National Institute of Agrobiological Sciences, 2-1-2 Kannondai, Tsukuba, Ibaraki 305-8602, Japan; ^2^Department of Biological Science and Technology, Science University of Tokyo, 2641 Yamazaki, Noda, Chiba 278-8510, Japan

## Abstract

The EPF family of plant TFIIIA-type zinc-finger (ZF) proteins (ZPTs) is characterized by long linkers separating ZF motifs. We previously reported that two-fingered ZPTs bind to two tandem core sites that are separated by several base pairs, each ZF making contact with one core site. Here we report further characterization of DNA-binding activities of ZPTs using four family members, ZPT2-14, ZPT2-7, ZPT2-8, and ZPT2-2, having inter-ZF linkers of different lengths and sequences, to investigate the correlation of the length and/or sequence of the linker with preference for the spacing between core sites in target DNAs. Selected and amplified binding site (SAAB)-imprinting assays and gel mobility shift assays prompted three conclusions. (1) The four ZPTs have common specificity for core binding sites—two AGT(G)/(C)ACTs separated by several nucleotides. (2) The four ZPTs prefer a spacing of 10 bases between the core sites, but each ZPT has its own preference for suboptimal spacing. (3) At a particular spacing, two zinc fingers may bind to the core sites on both strands. The results provide new information about how the diversity in linker length/sequence affects DNA-sequence recognition in this protein family.

## 1. Introduction

The EPF proteins form a subfamily of TFIIIA-type zinc-finger (ZF) proteins (ZPTs) of plants [1, 2]. The TFIIIA-type ZF motif is a sequence of CX_2–4_CX_3_FX_5_LX_2_HX_3–5_H, in which two cysteines and two histidines tetrahedrally coordinate a zinc atom to form a compact structure containing a *β*-hairpin and an *α*-helix (*ββα* motif), and the other conserved residues are packed to form a hydrophobic core [3–10]. Generally, in animals, multiple ZF motifs are present as tandem arrays linked by a conserved short sequence, the HC-link [11–13], and the ZF proteins interact with contiguous sets of triplet sequences, with each ZF making contact with 3–5 base pairs in the major groove of DNA. The ZPTs have 1, 2, 3, or 4 ZF motifs [14]. In most ZF motifs of ZPTs, a highly conserved sequence, QALGGH, is located within DNA-contacting surfaces [2, 14]. Since ZPT2-1 (renamed from EPF1) was first identified in petunia as a DNA-binding protein that interacts with a petal-specific promoter of the enolpyruvylshikimate-3-phosphate synthase gene [15], several EPF1-like ZPTs have been reported in various plant species [16, 17]. In *Arabidopsis*, a model plant whose genome has been sequenced, an estimated 64 ZF genes containing the QALGGH motif are encoded [16].

Two-fingered ZPTs have been implicated in various important regulatory processes. Some of these proteins are implicated in plant responses to abiotic stresses. These include petunia ZPT2-3 and various *Arabidopsis* proteins that are involved in drought tolerance [18, 19]. In addition, *Arabidopsis* ZPTs RHL41 [20] and SCOF1 [21] are implicated in plant responses to high-intensity light and low temperatures, respectively. The gene for petunia ZPT2-2 responds to various stress treatments such as drought, cold, salinity, and wounding [22]. Another two-fingered ZPT of petunia, MEZ1, is involved in the regulation of meiosis [23]. A vascular-bundle-associated ZPT, MsZPT2-1, is required for the formation of the central nitrogen-fixing zone of the root nodule in alfalfa [24]. Given that different ZPTs are implicated in various biological processes, different ZPTs most likely have different specificities for target sequences.

The ZPTs are characterized by long linkers between ZFs. Our previous study of DNA binding revealed that ZPT2-2 binds to two tandem core sites separated by several base pairs, each ZF making contact with one core site [25, 26]. We also found that a truncated peptide of ZPT2-2 can bind to multiple patterns of target sequences owing to the elasticity of the linker; an N-terminal finger (ZF1) was capable of binding to a core site located either upstream or downstream of the core site bound by the C-terminal finger (ZF2) [27]. The lengths of linkers between two adjacent ZF motifs vary among ZPTs, ranging from 19 to 65 amino acids, in contrast with an invariant length of linkers (5 amino acids, TGEKP) of cluster-type ZPTs in animals [14]. These characteristic features led us to speculate that the linkers in the ZPTs could play a role in the recognition of spacing between core binding sites [14, 26, 27].

In the present study, we characterized DNA-binding specificity of four ZPTs—ZPT2-7, ZPT2-14, ZPT2-8, and ZPT2-2—having inter-ZF linkers of various lengths and sequences, to further investigate the role of the linker in the recognition of target DNA sequences. Our results showed that the four ZPTs share common specificity for core binding sequences. However, the ZPTs showed different preferences for the spacing in target sequences. In addition, the results suggested that the ZPTs could bind to AGTs on either the same strand or both strands, dependent on the spacing contexts between the AGTs. The results provide new information about how the diversity in linker length/sequence affects DNA-sequence recognition in this protein family.

## 2. Experimental Procedures

### 2.1. Construction of Expression Vectors

To generate expression vectors for the production of truncated forms of ZPTs in *Escherichia coli*, we amplified DNA fragments of *ZPT2-7* (residues 32–114, ZPT2-7ZFB), *ZPT2-8* (residues 32–125, ZPT2-8ZFB), and *ZPT2-14* (residues 41–117, ZPT2-14ZF), encoding two zinc-finger motifs, by PCR using their full-length cDNAs as templates. Oligonucleotides used as primers for the PCR were primer 1 (5′-GGGTCTAGATTAGCACGTAAAATTTTCGAGTGCAAG-3) and primer 2 (5′-GGG CTGCAGTTACTTGTCATCACAATTTTTCTGCTTCTT-3′) for ZPT2-7ZFB, primer 1 and primer 3 (5′-GGG CTGCAGTTAAATTTCATCACAATTTTTCTGCTTCTG-3′) for ZPT2-8ZFB, and primer 4 (5′- GGG TCTAGATCTCCTAGTCGAGTTTTCGAGTGTAAA-3′) and primer 5 (5′-GGG CTGCAGTTACATCACAGCTCTATGCCTTCTCATATG-3′) for ZPT2-14ZF (restriction sites underlined). The PCR fragments were cut with *Xba*I and *Pst*I and inserted between the *Xba*I and *Pst*I sites of pMAL-(TEV)-ZPT2-2F12 [27], in place of the fragment encoding ZPT2-2F12, to yield pMAL-(TEV)-ZPT2-7ZFB, -ZPT2-8ZFB, and -ZPT2-14ZF. To generate an expression vector for the production of a truncated form of ZPT2-2 (residues 66–210, ZPT2-2ZF), we excised the corresponding DNA fragment from pMAL-ZPT2-2ZF [26] with *Xba*I and *Pst*I and inserted it between the *Xba*I and *Pst*I sites of pMAL-(TEV)-ZPT2-2F12 [27, 28] to yield pMAL-(TEV)-ZPT2-2ZF.

### 2.2. Expression and Purification of Recombinant Proteins

The expression vectors were introduced into *E. coli* strain JM109. The transformants were grown at 37°C, and protein production was induced for 3 h in the presence of 1 mM isopropyl-*β*-d-thiogalactopyranoside at 37°C. Cells were harvested and lysed by sonication in buffer A (20 mM Tris*·*HCl, pH 8.0, 10 *μ*M ZnCl_2_, 1 mM dithiothreitol [DTT], 1 mM phenylmethylsulfonyl fluoride [PMSF], and 0.05% Tween 20), and then centrifuged for 40 min at 20,000 xg. The resulting supernatants were loaded onto an amylose resin affinity column (New England Biolabs, Beverly, Mass, USA), washed with buffer B (20 mM Tris*·*HCl, pH 8.0, 10 *μ*M ZnCl_2_, and 0.5 mM DTT), and eluted with buffer B plus 10 mM maltose. The eluate was loaded onto a Hitrap Q column (GE Healthcare, Piscataway, NJ, USA) and eluted with a linear gradient of 0–1.0 M KCl in buffer B. The fractions (0.1–0.2 M KCl) were pooled, dialyzed against buffer B, and then digested with TEV protease (Invitrogen, Carlsbad, Calif, USA) for 10 h at 16°C in the presence of protease inhibitors (0.1 mM PMSF, 1 *μ*M pepstatin A, and 10 *μ*M leupeptin). The mixture was loaded onto a Protein-Pak SP 8HR AP mini column (Waters, Milford, Mass, USA) and eluted with a linear gradient of 0–1.0 M KCl in buffer B. The fractions (0.45–0.5 M KCl) were pooled and dialyzed against buffer C (25 mM HEPES-KOH, pH 7.6, 10 *μ*M ZnCl_2_, and 0.1 mM DTT).

### 2.3. SAAB-Imprinting Assay

DNA-binding sequences of ZPT2-14 were screened by SAAB-imprinting assay [28, 29] as described previously [27] using a library of randomly synthesized DNA (5′-GGCCTCGAGAAGCTT-(N)_25_-GGATCCTGCAGGGCC-3′) and PCR primers 5′-GGCCTCGAGAAGCTT-3′ and 5′-GGCCCTGCAGGATCC-3′. Purified ZPT2-14ZF proteins were incubated with ^32^P-end-labeled oligonucleotides containing a central stretch of 25-bp random sequences and separated by gel electrophoresis. Then ZPT2-14-bound oligonucleotides were recovered from the gel, amplified by PCR, and subjected to further rounds of selection. After 15 rounds of selection, electrophoretic patterns in gel mobility shift assays indicated that enrichment of ZPT2-14-binding DNA sequences had been saturated. ZPT2-14-bound oligonucleotides were recovered from the gel, amplified by PCR, and cloned (sequence pool after 15 rounds, SP_15_). We also cloned the oligonucleotides before selection by the SAAB-imprinting procedure (SP_0_). We sequenced 45 SP_15_ clones and 103 SP_0_ clones and counted the occurrence of various triplet sequences within the central 25-bp region. Enrichment (*X*) of each triplet through the selection was calculated as

(1) $$X=\;\frac{N_{15}}{T_{15}}-\frac{N_{0}}{T_{0}},$$

where *N*_15_ and *N*_0_ are total numbers of each triplet in the sequences in SP_15_ and SP_0_, respectively, and *T*_15_ and *T*_0_ are total numbers of sequenced clones, respectively. We regarded reverse complementary triplets as the same as the forward triplets.

### 2.4. DNA-Binding Assays

All binding reactions were carried out in 25 mM HEPES-KOH, pH 7.6, 40 mM KCl, 0.1% (v/v) Nonidet P-40, 10 *μ*M ZnCl_2_, 1 mg/mL bovine serum albumin, 10% glycerol, 10 *μ*g/mL double-stranded poly(dI-dC) (GE Healthcare), and 1 mM DTT. We mixed 10 000 cpm of ^32^P-end-labeled probe (at a concentration of approx. 1.5 × 10^–10^ M) with 1 × 10^–7^ M ZPT2-7ZFB, 1 × 10^–7^ M ZPT2-8ZFB, 3 × 10^–8^ M ZPT2-14ZF, or 3 × 10^–8^ M ZPT2-2ZF. After incubation for 25 min at 20°C, the mixtures were loaded into a 10% polyacrylamide gel (acrylamide/bisacrylamide, 29 : 1) and run in 0.5 × TB (45 mM Tris*·*borate, pH 8.0). The radiolabeled bands were visualized by autoradiography and quantitated using a STORM 840 system (GE Healthcare). Each experiment was performed four times using freshly thawed aliquots of peptides, unless otherwise indicated.

Apparent dissociation constants (*K*_*d*_) of ZPT2-14 to specific DNA sequences were determined as described in [30]. Probe DNAs were prepared by annealing two strands and purifying double-stranded DNA fractions in the 10% polyacrylamide gel. Gel mobility shift assays were performed using 1.510^−10^× M DNA and 1 × 10^−9^, 3 × 10^−9^, 1 × 10^−8^, 3 × 10^−8^, 1 × 10^−7^, 3 × 10^−7^, 1 × 10^−6^ M ZPT2-14 protein. *K*_*d*_ was calculated by plotting the ratio of free and bound DNA to the amounts of proteins used (*P*_0_) using the following equation:

(2) $$\frac{\text{free}\;\text{DNA}\;\left(\text{\%}\right)\;}{\text{bound}\;\text{DNA}\;\left(\text{\%}\right)}\;=\;K_{d}\cdot \frac{1}{\left[P_{0}\right]}.$$

## 3. Results

### 3.1. Inter-ZF Linkers of EPF-Family ZPTs Are Diverse in Length and Sequence

**Figure 1:**
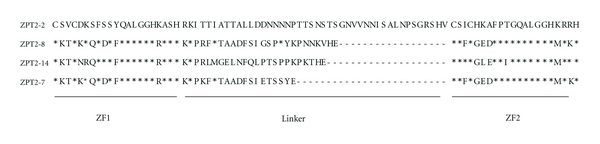
Amino-acid sequences in DNA-binding domains of four ZPTs: ZPT2-2 (AB000451), ZPT2-8 (AB006603), ZPT2-14 (AB006601), and ZPT2-7 (AB006602). Amino acids identical with those of ZPT2-2 are indicated by asterisks.

Amino-acid sequences in the ZF and inter-ZF linker regions of the four EPF-family ZPTs are shown in Figure 1. The ZF regions (ZF1 and ZF2) are well conserved among the four ZPTs. By contrast, the linker regions are very diverse in both length and amino-acid sequence. In contrast, the H-C links [13] are highly conserved in many TFIIIA-type ZF proteins and thus prompted us to investigate the significance of diverse linker length/sequence in DNA-sequence recognition by these ZPTs.

### 3.2. SAAB-Imprinting Assay of ZPT2-14

**Figure 2:**
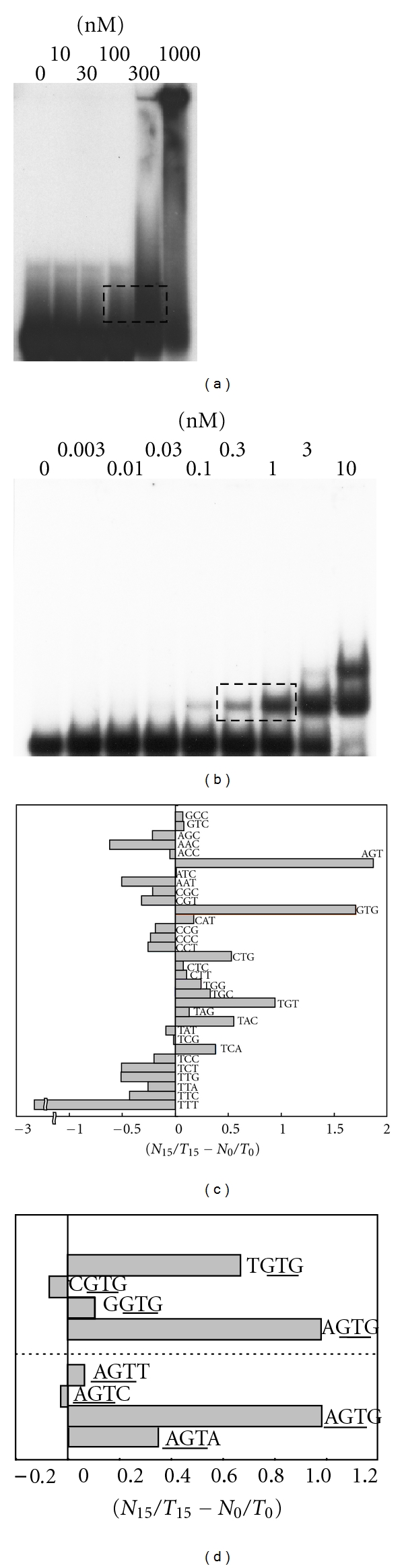
SAAB-imprinting assay of ZPT2-14-binding sequences. (a, b), Gel mobility shift assays after the first (a) and the fifteenth (b) rounds of selection by SAAB-imprinting procedure for ZPT2-14-binding sequence. Concentrations of ZPT2-14ZF in the reaction mixture are shown at the top of the lanes. Oligonucleotides were recovered from the shifted bands indicated by rectangles and processed. (c), Enrichment of respective triplets after 15 rounds of selection. Shown are increases in total numbers of each triplet occurring on both strands over those in the sequences before selection. (d), Enrichment of tetramers after 15 rounds of selection. Shown are increases in total numbers of tetramers including AGT or GTG over those in the sequences before selection.

As an initial step to study DNA-binding specificity of the four ZPTs, we first screened the DNA-binding sequences of ZPT2-14 by SAAB-imprinting assay using recombinant ZPT2-14 proteins. ZPT2-14-bound oligonucleotides after repeated selections by gel electrophoresis (Figures 2(a) and 2(b)) were sequenced. Inspection of the selected sequences by using 3-base windows revealed that AGT/ACT was most highly enriched by nearly 200% in the SP_15_ sequences relative to the SP_0_ sequences. GTG/CAC was enriched by almost as much. Other triplets were enriched by at most 100% or were underrepresented (Figure 2(c)). These two triplets, AGT/ACT and GTG/CAC, are overlapping portions of a tetramer, AGTG/CACT. The inspection of the selected sequences by using 4-base windows revealed that AGTG/CACT was significantly enriched over other tetramers including AGT or GTG, as indicated in Figure 2(d).

**Figure 3:**
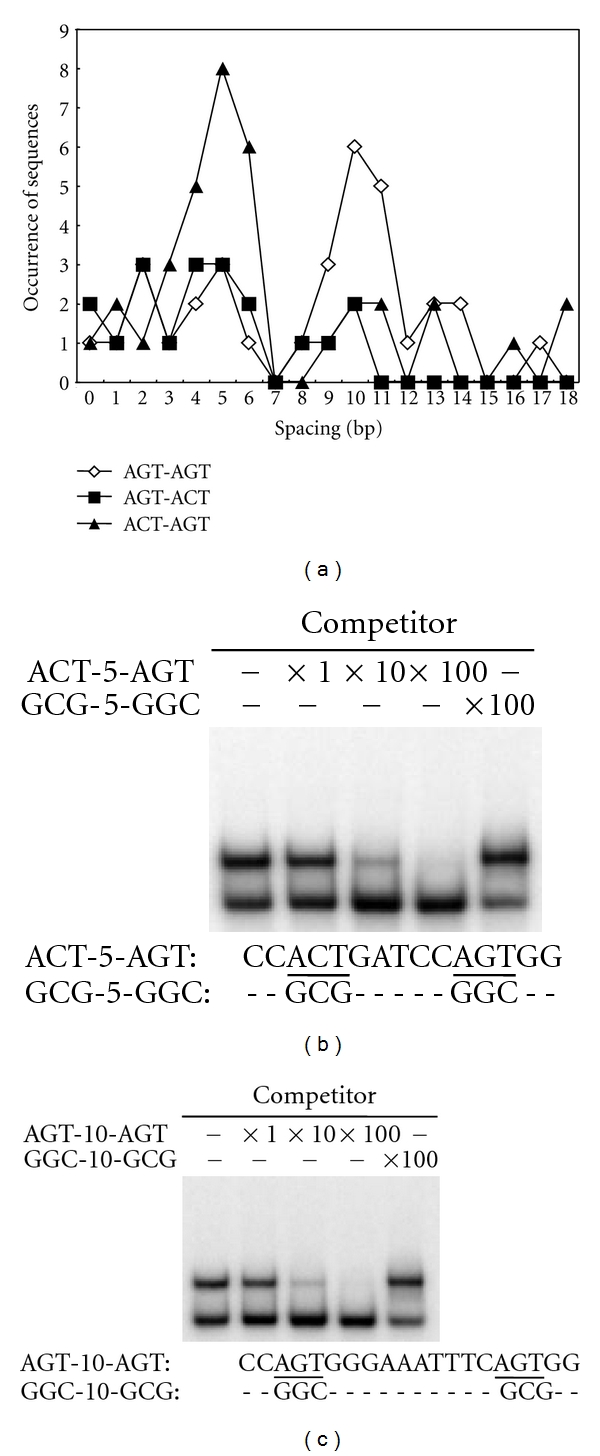
ZPT2-14 binds to two core sequences separated by a particular spacing. (a), Distribution of spacing between two AGT/ACTs in the ZPT2-14-binding sequences selected by SAAB imprint assay (in AGT-AGT, AGT-ACT, and ACT-AGT configurations). Total of the three configurations are also shown. (b, c), Competition analysis of the ZPT2-14-binding sequences in gel mobility shift assays. ^32^P-end-labeled probes with 5-base spacing—ACT-5-AGT (b)—or 10-base spacing—AGT-10-AGT (c)—were incubated with ZPT2-14ZF in the presence or absence of double-stranded competitors of ACT-5-AGT or GCG-5-GGC sequences in standard reaction mixtures. The amounts of competitors used were 0.2 ng (×1), 2.0 ng (×10), and 20 ng (×100). Central sequences of the probes and competitors are shown under the panels, with putative core sites underlined. Sequences of the probe DNA flanking the central regions are 5CTCGAGAAATTTCCCGGAATT-central region-CCGGAATTTCCCGGGGGGATCC-3′. The competition experiments were done twice and gave similar results.

Of the 45 clones sequenced, 42 included multiple AGT/ACT triplets in the central 25-bp region. Thus, we investigated the spacing between the pairs of AGT/ACT triplets that occur in three patterns (AGT-AGT, AGT-ACT, and ACT-AGT; Figure 3(a)) in the SP_15_ sequences. We observed two peaks of spacing in all three patterns, with the 7-base spacing missing. One peak was at the spacing of 5 bases, where 5′-ACT-N_5_-AGT-3′ was predominant. The other peak was at the spacing of 10 bases, where 5′-AGT-N_10_-AGT-3′ was predominant. To examine whether these two patterns of sequences, having AGT or ACT at different spacings, permit sequence-specific binding of ZPT2-14, we performed competition assays using two probes, ACT-5-AGT and AGT-10-AGT (Figures 3(b) and 3(c)). The binding of ZPT2-14 to both probes was clearly outcompeted by excess amounts of competitors with identical sequences as probes, but not by competitors having mutations in AGT/ACTs. These results confirm the binding of ZPT2-14 to AGT/ACTs as core sites in different spacing contexts.

### 3.3. Sequence Specificity of ZPT2-14 for Core Binding Sites

**Figure 4:**
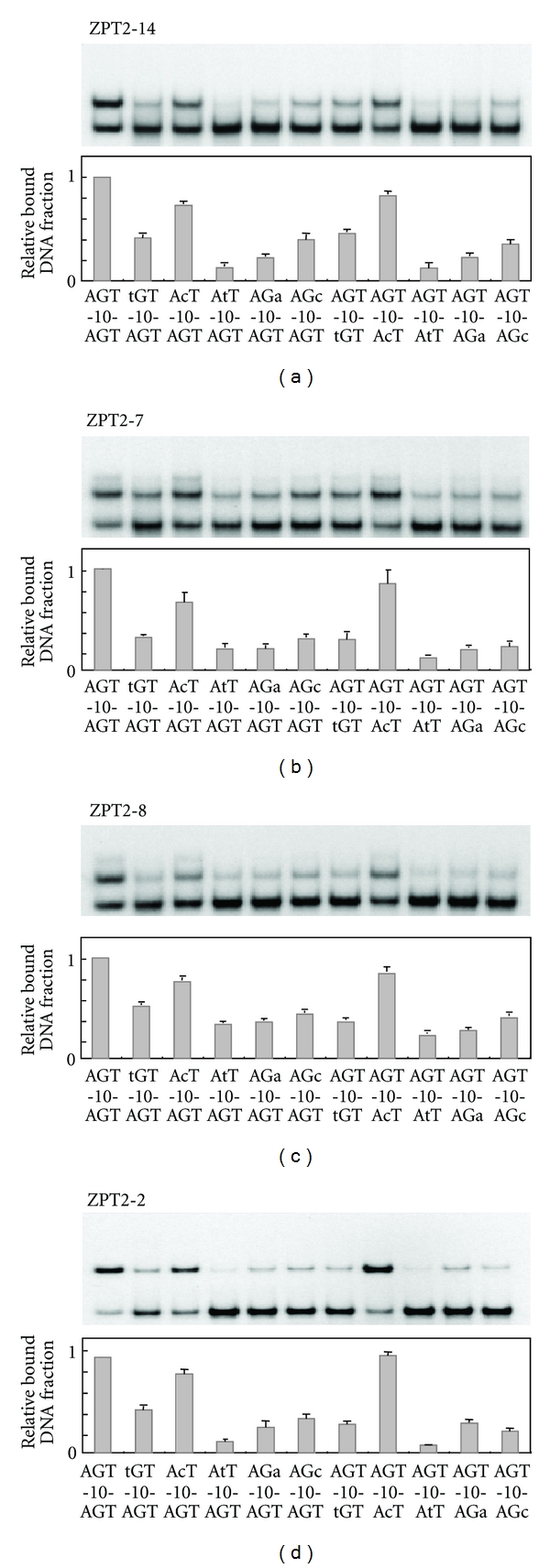
Specificity for core binding sequences of four ZPTs. Four ZPT peptides—ZPT2-14ZF (a), ZPT2-7ZFB (b), ZPT2-8ZFB (c), and ZPT2-2ZF (d)—were tested for binding activities to AGT-10-AGT and its mutant sequences with one-base substitutions in the core sites. Fractions of bound DNA are shown as relative values to the bound fraction for AGT-10-AGT, with representative electrophoretic patterns above them. The data represent averages of four experiments, with bars representing standard deviations. The probes are essentially the same as AGT-10-AGT in Figure 3 except for the mutated bases as indicated by lowercase letters.

**Figure 5:**
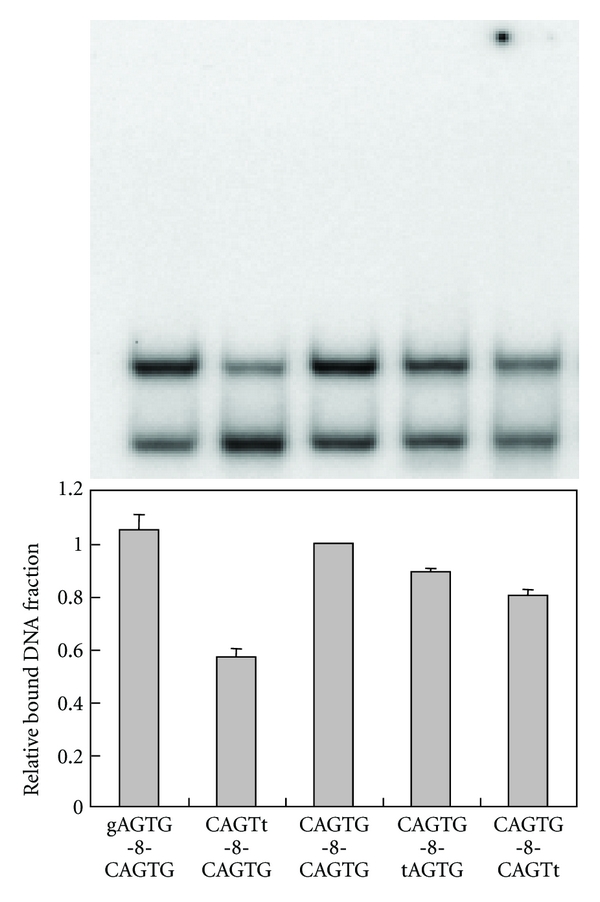
Preference for the sequences around AGT core sites. ZPT2-14ZF was tested for binding to a set of probes having one-base mutations outside the AGT core sites. Fractions of bound DNA are shown as relative values to the bound fraction for AGT-10-AGT, with representative electrophoretic patterns above them. The data are averages of four experiments, with bars representing standard deviations. The probes are essentially the same as AGT-10-AGT in Figure 3 except for the mutated bases as indicated by lowercase letters.

**Figure 6:**
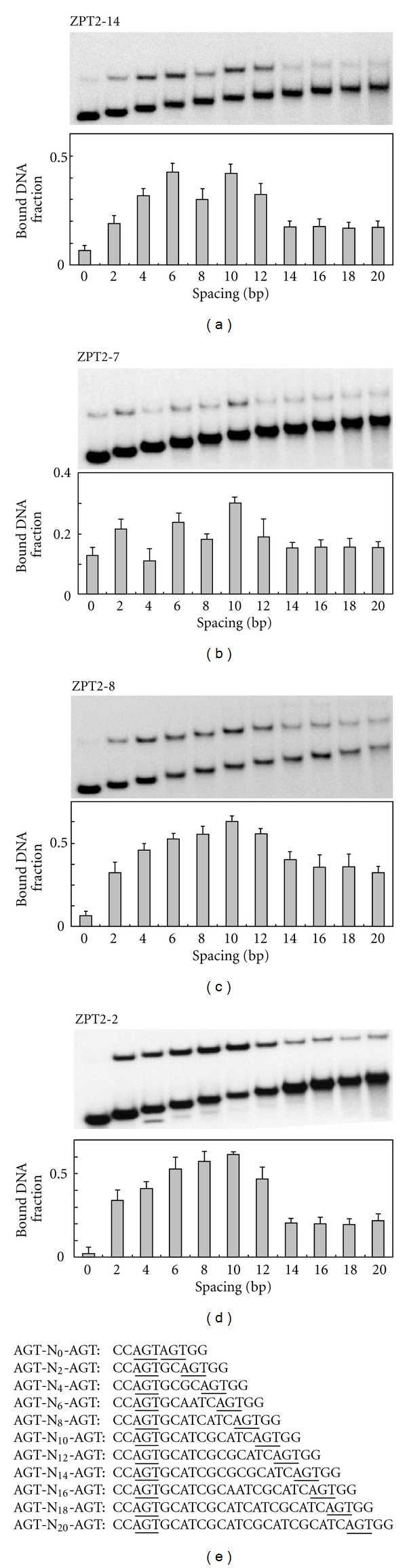
Recognition of spacing in target sequences. (a)*–*(d), DNA binding of four ZPT peptides—ZPT2-14ZF (a), ZPT2-7ZFB (b), ZPT2-8ZFB (c), and ZPT2-2ZF (d)—to DNA sequences with various lengths of spacing between two core sequences. Fractions of bound DNA are shown with representative electrophoretic patterns above them. The data are averages of four experiments, with bars representing standard deviation. (e) Sequences of the central regions of probes. Two core binding sites (AGT) in each probe are underlined. Besides the core sites, AGT/ACT is excluded from these regions, and AT/GC contents were balanced in these regions. Sequences flanking the central regions are 5′-CTCGAGAAATTCC-central region-GGCCGGGGGGATCC-3′.

We further investigated the sequence specificity of ZPT2-14 for the core binding sites by using a set of probes having one-base substitutions in the core triplets in the AGT-10-AGT probe (Figure 4(a)). AGT-10-AGT probe showed highest affinities to ZPT2-14 among all the probes. ACT-10-AGT and AGT-10-ACT probes showed only slightly lower affinities, but other mutant probes showed markedly reduced binding affinities. Thus, 5′-AGT-N_10_-AGT-3′ is an optimal ZPT2-14-binding sequence, and the replacement of one of the two AGTs with ACT caused only minor effect on ZPT2-14-binding affinity. Thus, we used AGT-N-AGT probes for further analysis as a representative. To investigate the effects of sequence contexts around the AGT core triplets, we tested a set of probes (*x*_1_AGT*y*_1_-8-*x*_2_AGT*y*_2_) having one-base mutations outside the two AGT core sites (Figure 5). The binding assays revealed a preference for G at the positions downstream of AGT (*y*_1_ and *y*_2_), with the preference particularly strong at *y*_1_, but did not reveal any preference for the bases upstream of AGT (*x*_1_ and *x*_2_). However, the effects of base substitution at these positions were minor compared with those in the AGT core sites (Figures 4(a) and 5). Collectively, these results demonstrate that the core binding sites of ZPT2-14 are two AGT(G)/(C)ACTs. This conclusion is in accordance with the tetramer sequence enriched in the SAAB-imprinting assays, and we used this consensus sequence in the basic probes used in this work (Figures 3, 4, and 6).

### 3.4. ZPT2-14 Binds to Two Sequences Differing in Both Core Sequences and Spacing

The SAAB-imprinting assays using ZPT2-14 described above revealed two candidate target sequences, 5′-AGT-N_10_-AGT-3′ (=5′-AGTG-N_9_-AGTG-3′) and 5′-ACT-N_5_-AGT-3′ (=5′-CACT-N_5_-AGTG-3′) (Figure 3). The two sequences differ in both core sequences and spacing between the core sequences, suggesting that ZPT2-14 prefers different combinations of core sequences under different spacing contexts. We determined apparent dissociation constants (*K*_*d*_) for the binding of ZPT2-14 to 6 sequences that include 5- and 10-base spacings between core binding sites with different triplet sequences (Table 1). Of the 3 sequences with 10-base spacings, ZPT2-14 showed smallest *K*_*d*_ to AGT-10-AGT and slightly larger *K*_*d*_s to AGT-10-ACT and ACT-10-AGT sequences, consistent with the results in Figure 4(a). By contrast, of the 3 sequences with 5-base spacings, ZPT2-14 showed obviously larger *K*_*d*_ to AGT-5-ACT compared with those to 2 other sequences, AGT-5-AGT and ACT-5-AGT. Thus, the spacings between core sites largely affect the preference for core triplet sequences.

### 3.5. ZPT2-14, ZPT2-7, ZPT2-8, and ZPT2-2 Recognize Common Core Sequences

In our previous study, 5′-AGT-N_10_-AGT-3′, one of the sequences binding with high affinity to ZPT2-14, also showed high affinity for ZPT2-2 [26]. To test whether this sequence binds with high affinity to other ZPTs as well, we tested ZPT2-7, ZPT2-8, and ZPT2-2 for binding to AGT-10-AGT and mutant probes with substitutions in the core sites (Figure 4). Gel mobility shift assays revealed that all three ZPTs bound with high affinity to AGT-10-AGT, with similar effects of mutations in the core sites to those observed with ZPT2-14. These results indicate that the four ZPTs share common sequence specificity for core binding sites. We previously reported an optimal binding sequence of ZPT2-2 N-terminal ZF to be AGC(T) [27]. This inconsistency could be due to the use of further truncated ZPT2-2 peptide in the previous experiments.

### 3.6. Recognition of Spacing between Core Binding Sites

The results of SAAB-imprinting assays indicate that ZPT2-14 prefers a specific spacing between core binding sites, as does ZPT2-2 [26, 27]. This raises the questions of whether each ZPT has its own preference for the spacing in target sequences, and, if so, how the length/sequence of linkers differing among different ZPTs is correlated with the preference for the spacing. To answer these questions, we compared the preferences of the four ZPTs for spacing in target sequences by using a set of probes with various spacings between two AGTs (AGT-N*x*-AGT, *x* = 0–20 bases) (Figure 6(e)). Gel mobility shift assays revealed that ZPT2-14, having a linker of 24 amino acids long, showed two peaks of binding affinity, at the spacings of 6 and 10 bases (Figure 6(a)). ZPT2-7, having a linker of 19 amino acids long, showed a peak of the highest affinity at 10-base spacing, and two additional peaks of suboptimal affinity at the spacings of 2 and 6 bases (Figure 6(b)). In contrast, ZPT2-8 and ZPT2-2, having relatively long linkers of 26 and 44 amino acids long, respectively, showed only one broad peak of spacing preference, with maximum affinity at 10 bases (Figures 6(c) and 6(d)). In summary, the four ZPTs all preferred the spacing of 10 bases, but those having relatively short linkers tended to show multiple peaks of preferred spacing. Thus, the diversity in length/sequence of inter-ZF linkers does influence the preference for the spacing in target DNA sequences.

## 4. Discussion

**Figure 7:**
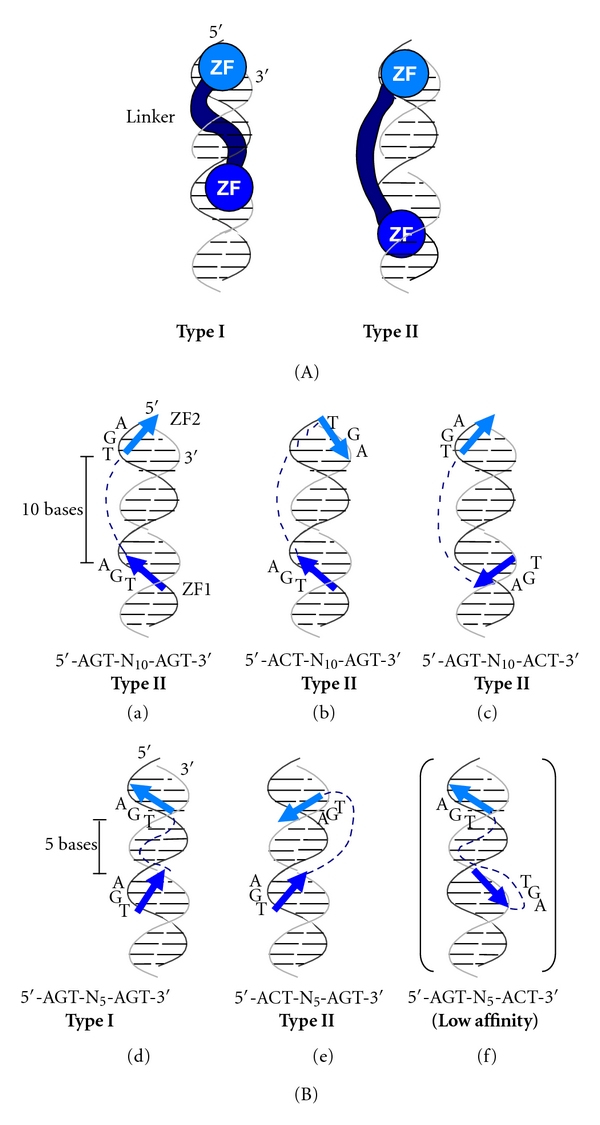
Proposed DNA-binding modes of ZPTs. (A) Two types of ZPT–DNA interactions. In Type-I binding (left), the linker wraps around the major groove. In Type-II binding (right), the linker traverses the minor groove. (B) DNA-binding models of ZPT2-14 in different spacing contexts. In 10-base spacing context (5′-AGT-N_10_-AGT-3′), two ZFs bind to AGTs on either strand presumably by Type-II mode (a)–(c). In 5-base spacing context, ZPT2-14 binds to 5′-AGT-N_5_-AGT-3′  in Type I mode (d). It also binds to 5′-ACT-N_5_-AGT-3′ in antiparallel Type II mode at high affinity (e). Binding to 5′-ACT-N_5_-AGT-3′ is at low affinity (f)*.*

In this study, we characterized the DNA-binding properties of four ZPTs with diverse length and/or sequence of inter-ZF linkers, aiming at investigating how they are correlated with the preference for spacing in target DNA sequences. The four ZPTs, although differing in the length and sequence of inter-ZF linkers, had common specificities for core binding sites, which enabled us to compare their spacing preference in a simple system using the same set of probes. All four ZPTs preferred the spacing of 10 bases but showed various patterns in their preference for suboptimal spacing lengths in the range of less than 10 bases (Figure 6). We attempt below to explain the differential preference for the spacing by assuming two types of ZPT-DNA interaction (Types I and II), as shown in Figure 7(A). In Type-I interaction, the linker wraps around the major groove of a DNA helix. In Type-II interaction, the linker crosses over the minor groove. In Type-II interaction, the helical periodicity of the DNA helix (approximately 10.5 base pairs in the B-form helix) is a crucial factor determining the spacing preference, because 2 zinc fingers make contact with DNA from the same side of the DNA helix in Type-II interaction. In Figure 6, ZPT2-14 showed two peaks of binding affinity to AGT-N*x*-AGT sequences with shorter (6 bases) and longer (10 bases) spacings. On the basis of the consideration described above, the peak at 6-base spacing is presumably in part due to Type-I binding. Presumably, the peak at 10-base spacing is mostly due to Type-II binding, because the linker is not long enough for binding in Type I mode. ZPT2-7 showed two peaks of suboptimal binding at spacings of 2 and 6 bases, in addition to a main peak at 10-base spacing. In this case, essentially the same interpretation as that given for ZPT2-14 also holds for the peaks at 6- and 10-base spacings. The peak of suboptimal binding at 2-base spacing could be due to an alternative stable structure of ZPT2-7 that is defined by the relatively short linker of this ZPT. ZPT2-2 and ZPT2-8 showed broad peaks without minor peaks of suboptimal binding. Presumably, the longer linkers of these ZPTs permit more flexible binding than those of ZPT2-14 and ZPT2-7, making these ZPTs less sensitive to steric restrictions upon DNA binding associated with different spacings.

On the basis of the results in Table 1, we propose that the two ZFs in ZPTs can bind to two core sites in both parallel and antiparallel orientations depending on spacing contexts. In the 10-bp spacing context, ZPT2-14 binds to 5′-AGT-N_10_-AGT-3′, 5′-ACT-N_10_-AGT-3′, and 5′-AGT-N_10_-ACT-3′ at similar affinities (Figure 4 and Table 1). In this case, the two ZFs presumably bind to two core sites in the same or opposite orientations presumably by the Type-II mode (Figure 7(B), a–c). By contrast, in the 5-bp spacing context, ZPT2-14 showed high affinities to 5′-AGT-N_5_-AGT-3′ and 5′-ACT-N_5_-AGT-3′, but lower one to 5′-AGT-N_5_-ACT-3′ (Table 1). We speculate that, in the 5-bp spacing context, ZPT2-14 binds to 5′-AGT-N_5_-AGT-3′ in Type I mode by interacting with two AGTs in the same orientations (Figure 7(B)-d). ZPT2-14 binds to 5′-ACT-N_5_-AGT-3′ in antiparallel Type II mode with each finger binding to core sites in opposite orientations (ACT is reverse complementary to AGT, Figure 7(B)-e). ZPT2-14 also binds to 5′-AGT-N_5_-ACT-3′ with each finger binding to core sites in opposite orientations, but with much lower affinity. The low affinity is presumably because of suboptimal fitting of ZFs with core sites due to steric constraint (Figure 7(B)-f). This interpretation is inline with our previous observation that multiple arrangements of two core sites accommodate binding of truncated ZPT2-2 peptides [27]. The binding to the sequences in both strands has also been reported for *δ*EF1 and SIP1, vertebrate two-handed ZPTs that have long linkers between ZFs [31]. Presumably, this unique mode of protein-DNA interaction is characteristic of those plant and vertebrate ZPTs having long inter-ZF linkers.

The present study showed that EPF-family ZPTs have largely common specificity for the core sequences in target DNAs and that these proteins show some variations in preference for the spacing in target sequences. Although the variations were minor in our *in vitro* assay conditions, they could have more pronounced effects *in vivo* and could serve as determinants of the selectivity of target genes by each ZPT.

## Figures and Tables

**Table 1:** Apparent dissociation constants for the binding of ZPT2-14 to target sequences with 5- and 10-base spacings. Apparent dissociation constants of ZPT2-14ZF to two types of sequences with different spacing were determined as described in experimental procedures. The data are averages of four experiments, with bars representing standard deviations. The probes are essentially the same as AGT-10-AGT and ACT-5-AGT sequences shown in Figure 3 except for the mutated bases.

Probe	*K*_*d*_ (nM)
AGT-10-AGT	8.0 ± 0.3
AGT-10-ACT	10.4 ± 1.6
ACT-10-AGT	11.6 ± 1.9
AGT-5-AGT	9.1 ± 1.2
AGT-5-ACT	21.7 ± 3.4
ACT-5-AGT	7.3 ± 0.7

## Abbreviations

ZF: Zinc finger
ZPT: Zinc-finger protein of TFIIIA type
SAAB: Selected and amplified binding site
PCR: Polymerase chain reaction
TEV: Tobacco etched virus
DTT: Dithiothreitol
PMSF: Phenylmethylsulfonyl fluoride.
